# Carcinogenicity assessment: Addressing the challenges of cancer and chemicals in the environment

**DOI:** 10.1016/j.envint.2019.04.067

**Published:** 2019-07

**Authors:** Federica Madia, Andrew Worth, Maurice Whelan, Raffaella Corvi

**Affiliations:** European Commission, Joint Research Centre (JRC), Ispra, Italy

**Keywords:** Carcinogenicity testing, Environmental health, Chemical exposure, Cancer risk, REACH

## Abstract

Cancer is a key public health concern, being the second leading cause of worldwide morbidity and mortality after cardiovascular diseases. At the global level, cancer prevalence, incidence and mortality rates are increasing. These trends are not fully explained by a growing and ageing population: with marked regional and socioeconomic disparities, lifestyle factors, the resources dedicated to preventive medicine, and the occupational and environmental control of hazardous chemicals all playing a role. While it is difficult to establish the contribution of chemical exposure to the societal burden of cancer, a number of measures can be taken to better assess the carcinogenic properties of chemicals and manage their risks. This paper discusses how these measures can be informed not only by the traditional data streams of regulatory toxicology, but also by using new toxicological assessment methods, along with indicators of public health status based on biomonitoring. These diverse evidence streams have the potential to form the basis of an integrated and more effective approach to cancer prevention.

## Highlights

• Cancer has become a key public health concern worldwide and at EU level.
• Cancer disease is a central priority of EU public health policy.
• Public health policy actions cannot be decoupled from environmental policy actions.
• Carcinogenicity assessment must evolve to ensure adequate levels of human protection.
• The regulatory assessment of chemical toxicity should consider human specific cancers.

## Graphical abstract

**Opportunities for carcinogenicity assessment to address the challenges of cancer disease and chemicals in the environment**. The safety assessment of carcinogenicity needs to evolve to keep pace with changes in the chemical environment and cancer epidemiology. Future strategies for assessing carcinogenicity based on a more holistic approach, can take into account the prevalence of certain cancers, the contribution to the disease of different risk factors, the study of relationships between chemical exposures and risk factors, the disease aetiology and links with other disorders. In addition, changes in chemical exposure patterns and exposed populations are also critical considerations.

**Figure:**
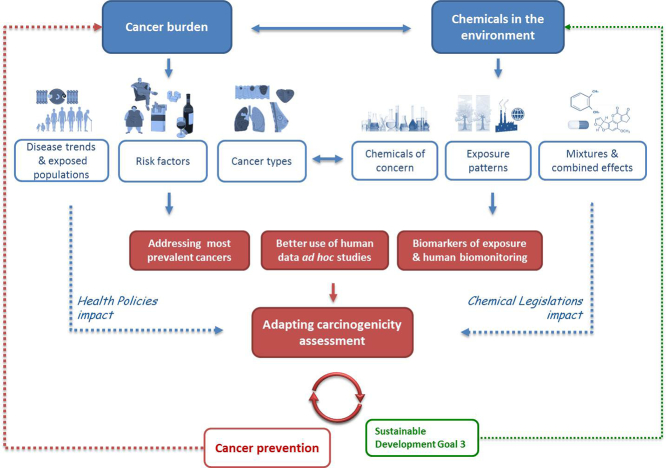

## 1 Introduction

**Fig. 1:**
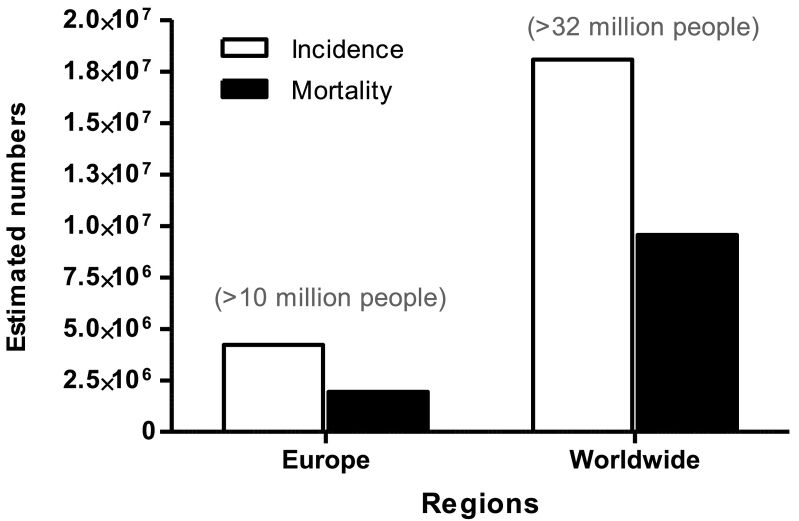
Cancer incidence and mortality in 2018. European and Global cancer incidence and mortality figures. Data are reported as estimated number of incident cases and deaths for all cancers (both sexes, all ages). Data refer to year 2018. In parenthesis, estimated prevalence (5-year survival from first diagnosis). (Global Cancer Observatory, http://gco.iarc.fr/today/home, and ECIS, https://ecis.jrc.ec.europa.eu/index.php, accessed on 06/11/2018 @ European Union 2018).

Cancer has become a significant public health concern, emerging worldwide as the second leading cause of morbidity and mortality among non-communicable diseases after cardiovascular diseases. Global estimates for 2018, which cover 36 different cancers in 185 countries, report 18.1 million new cancer cases and approximately 9.6 million cancer-related deaths (Fig. 1) (Bray et al., 2018). The number of new cancer cases is projected to increase to 24.1 million annually by 2030 and to 29.5 million by 2040. In addition, >32 million people are living with a cancer diagnosis (5-year prevalence), thus contributing heavily to the toll on health systems (Ferlay et al., 2018; Global Cancer Observatory, 2018). Population growth and ageing can only partly explain the rising figures in incidence and mortality. A number of factors account for the increasing global burden of the disease and regional disparities in tumour types. These factors are primarily associated with each country's level of socioeconomic development and therefore include life styles, hygiene levels, environmental pollution, spread of communicable diseases (e.g. HIV, HPV, or hepatitis B), and the economic resources dedicated to preventive medicine and occupational exposure control measures (Bray et al., 2012; Ferlay et al., 2013; Jing et al., 2014; WHO Cancer Report, 2014). Nearly half of all new cancers estimated for 2018 are in Asia, where 60% of the population lives. Europe accounts for 23.4% of total cancer cases although it is home to only 9% of the global population (Bray et al., 2018). In fact, estimated rates of new cases (adjusted per population size and age) are highest in more industrialised regions (ECIS, 2018; Global Cancer Observatory, 2018; WHO Cancer Report, 2014). The greatest impact in terms of mortality is in low- and middle-income countries or those experiencing significant industrial growth, many of which are ill-equipped to cope with the escalating burden and cost of the disease (Bray et al., 2018; WHO Cancer Report, 2014).

In Europe, cancer is the most frequently occurring form of non-communicable disease and the second most common cause of death, after cardiovascular diseases. Estimates for 2018 indicate 4.2 million new cancer cases and 1.94 million cancer deaths, as reported by the European Cancer Information System (Fig. 1): a much higher rate on a per capita basis than the global figure (ECIS, 2018; Bray et al., 2018; Global Cancer Observatory, 2018). This is in spite of major improvements in diagnosis and therapy, and a slight decrease in deaths linked to certain cancers.

Furthermore, 10 million people are living with a cancer within 5 years of being diagnosed (Arnold et al., 2015; Global Cancer Observatory, 2018). A number of interrelated causes contribute to the high cancer incidence in Europe. In particular, these include: 1) lifestyles typical of industrialised countries (high very high HDI) (Colditz and Wei, 2012; Landrigan et al., 2016; WHO Cancer Report, 2014); 2) high urbanisation and dense distribution of aged population, with long-term exposure to occupational and environmental carcinogens and medicines (EEA Chemicals for a sustainable future, 2018); 3) chronic exposure to particulate matter, ozone, benzo[*a*]pyrene and other pollutants that are above European standard limits and WHO air quality guidelines which have been linked to significant increase of respiratory NCD and cancers (EEA Air quality, 2018); 4) the deployment of early detection and screening programs (e.g. prostate or thyroid cancer) that contribute to a documented increase of cancer incidence due to the detection of precursor lesions (Jönsson et al., 2016; Luengo-Fernandez et al., 2013; Vineis and Wild, 2014).

Occurrence and survival data show significant disparities in tumour types across European countries; these can be linked to differences in environmental and occupational exposure, including air pollution level differences, lifestyles, demographic factors and to some extent to the budget that national governments devote to health care (Arnold et al., 2015; ECIS, 2018; Jönsson et al., 2016; Luengo-Fernandez et al., 2013; Vineis and Wild, 2014). For example, Denmark has one of the highest incidence rates but spends less on cancer than Sweden, which has lower incidence rates (Jönsson et al., 2016).

At international level, the World Health Organization (WHO) has recognised the burden of cancer on health and the resulting social and economic impacts. In 2017, the 70th World Health Assembly of the WHO adopted a resolution on cancer prevention and control, with a broad consensus that “*Cancer is a growing public health concern which requires increased attention*, *prioritization and funding*” (WHO Assembly Resolution, 2017). The resolution “*urges Member States*, […], *to implement comprehensive cancer prevention and control programs*, *including management of disease* …[…] *fostering the development of effective and affordable new cancer medicines*” but also, *to enhance the coordination of activities related to the assessments of hazards and risks and the communication of those assessments* (WHO Assembly Resolution, 2017).

Within the United Nations (UN), the Sustainable Development Goals (SDGs) of the 2030 UN Agenda for Sustainable Development are also relevant. In particular, SDG-3 aims “[…] *to ensure healthy lives and promote well*-*being for all at all ages*”, includes specific targets to reduce premature mortality from non-communicable diseases by one third and “[…] *to substantially reduce the number of deaths and illnesses from hazardous chemicals and air*, *water and soil pollution and contamination*” (United Nations, 2015).

Cancer disease is a central priority of EU health policy and a number of initiatives on screening, control and prevention programs are ongoing to reach a 15% reduction of cancer incidence by 2020 and to target 2030 SDGs (EU Communication, 2016). Several initiatives have been put in place over the past two decades following a recommendation of the Council on cancer screening (EU Council Recommendation, 2003) and the establishment of a European Partnership to support the Member States in their efforts in fighting cancer (ECIS, 2018; EU Commission Communication, 2009; EU Parliament Resolution, 2008).

Acknowledging the important contribution that effective chemical safety assessment has to inform risk management measures and reduce the burden of cancer, we reflect here on the role of carcinogenicity assessment in the broader public health context. We take into consideration actual cancer scenarios, the contribution of chemical exposure to the disease, the impact of current EU legislative measures, and the influence of public health policies. In addition, we report on current scientific advances in carcinogenicity assessment and their potential to help the fight against cancer.

## 2 Risk factors and cancers of most concern

Cancer is a broad term encompassing many different highly heterogeneous but related diseases affecting potentially almost every tissue in the body (NIH NCI, 2018). While different explanations have been put forward for the causes and mechanisms of cancer, it is acknowledged that there is a complex interplay of multiple risk factors, which can contribute at the same time or at different stages over longer time frames (Anand et al., 2008).

From an evolutionary perspective, cancer can be regarded as a conserved trait across species, typically the result of an adaptive response to rapid changes in the environment (Aktipis and Nesse, 2013). From this perspective, the ecological context of cancer cells parallels that of the organisms they live in. They respond similarly to: the emergence of new stressors; to increased availability of nutrients; to the allocation of energy to growth at the expense of survival (as reproduction at the expense of health); to cellular defence mechanisms (e.g. action of the immune system); and to the co-evolution with pathogens (Aktipis et al., 2013; Lichtenstein, 2005).

While cancers cannot be completely avoided, evidence strongly suggests that susceptibility to the disease can be reduced significantly by reducing the impact of several risk factors.

Cancer risk factors that might be largely preventable include biological agents (infections), exposure to synthetic chemicals through work or consumer products, and lifestyle factors such as exposure to sunlight, poor diet, being overweight, tobacco use and consumption of alcohol. These risk factors are reported to collectively contribute to the development of 70–95% of all cancers (Colditz and Wei, 2012; Wu et al., 2016). While the specific contribution from chemicals to cancer is difficult to quantify with certainty, a number of estimates have been made. In 2008, Anand and colleagues (Anand et al., 2008), reported the following relative contributions: diet (30–35%); tobacco (25–30%); infections (15–20%); obesity (10–20%); alcohol (4–6%); others, including pollutants and radiation (10–15%). Similar estimates were reported by Belpomme and colleagues and WHO (Belpomme et al., 2007; WHO Cancer Report, 2014). Colditz and Wei, excluding tobacco use, proposed that the contribution from chemicals is 4–10% (Colditz and Wei, 2012).

Family history and ageing represent instead unavoidable risk factors. For 5–10% of cancer cases, significant correlations with specific inherited genes have been identified (e.g. BRCA1 and BRCA2 gene mutations in specific breast cancer types; HPC1 and TLRs in prostate cancer; MLH1, MSH2, MSH6, APC, PTEN in colorectal cancer) (Colditz and Wei, 2012; Wu et al., 2016). With increasing age, stress resistance decreases as well as the ability to repair cellular and DNA damage. Since this is combined with the cumulative use of pharmaceuticals and exposure to stressors, including chemicals, the vulnerability to cancer might increase with age. For some types of cancer, such as oesophageal carcinoma, liver hepatocellular carcinoma, pancreatic adenocarcinoma, pheochromocytoma, stomach adenocarcinoma, bladder and colon cancers, the effect of ageing has been suggested to outweigh other risk factors (Podolskiy and Gladyshev, 2016).

Despite the high heterogeneity of cancer types, humans are mainly afflicted by breast, prostate, lung and colorectal cancer. There are also increasing trends in cancers of the stomach, cervix, liver and bladder (Rahib et al., 2014; WHO Cancer Report, 2014).

**Fig. 2:**
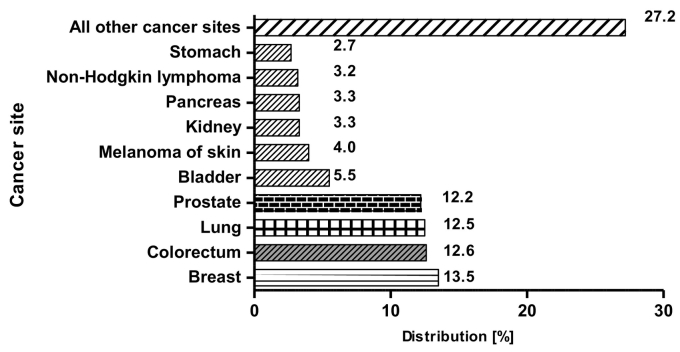
Estimated incidence by cancer site in EU in 2018. The chart reports the estimated percentage distribution of cancers in the EU (28 Member States) for the year 2018, both sexes, all ages. Each bar is proportional to the contribution of each cancer to the total.

Source: ECIS https://ecis.jrc.ec.europa.eu, accessed on 03/15/2019 © European Union, 2019.

In Europe, breast cancer is the most common, with nearly half a million new cases per year. This is closely followed by colorectal, lung and prostate cancers, with lung cancer showing the poorest prognosis and leading to approximately 20% of all cancer deaths. Prostate cancer has shown a levelling-off in its mortality rate which is most likely due to the introduction of early screening for the prostate-specific antigen (PSA) biomarker (Fig. 2) (ECIS, 2018; Heijnsdijk et al., 2018; Torre et al., 2015).

The prevalence of these four cancers is not attributable to a single cause although they share some common traits. Notably, these cancers have similar anatomical origin. In fact, their cells derive from the same original epithelial cell type (same original germ layer). Epithelial cancers (carcinomas) represent 80–90% of all cancers, which is not surprising since epithelial tissues are the most abundant in the body (McCaffrey and Macara, 2011). Since epithelia lie at the interface between the organism and its environment, they are the first to respond to any type of insult. Such responses occur in a multi-step process and may lead to chronic inflammatory pathologies and cancer formation, as observed in the case of colorectal cancer in the gastrointestinal tract (Madia et al., 2012).

Although the anatomical origin can influence tumour classification and development, similarities in mutations and signalling pathways have been observed. These have been linked to family history or specific mutations as in the case of BRCA prostate and breast cancers (Agalliu et al., 2009; Rimar et al., 2017; Song et al., 2017) and to common underlying mechanisms and signalling pathways (e.g. PI-3-Kinase/Akt, RTK-RAS, etc.) that are shared across the different cancers (Hoadley et al., 2018; Thorsson et al., 2018).

Furthermore, compelling evidence has recently linked breast, prostate and colorectal cancers with insulin resistance. This is a pathologic condition observed in metabolic disorders such as obesity and type-2 diabetes mellitus and associated immune deregulation to which both genetic and environmental factors might jointly contribute.

The environmental factors likely reflect the shift toward unhealthy dietary habits typical of industrialised countries, including overeating and consumption of processed food or excessive amounts of nutrient supplements, hormones and growth factors. In addition, exposure to contaminants in the food chain may play a role (Arcidiacono et al., 2012; Djiogue et al., 2013; Fontana and Partridge, 2015; Persano et al., 2015; Tosti et al., 2018; WHO Cancer Report, 2014).

The prevalence of lung cancer is attributable mainly to occupational exposures but also to air pollution and tobacco use. A higher risk of lung cancer has also been associated with chronic pulmonary diseases (Tu et al., 2017).

## 3 Chemicals and cancer

Scientific research has led to considerable insights into the many ways exogenous chemicals can adversely affect human health and cause cancer. However, accurately estimating the proportion of all cancer risk attributable to chemical exposure remains a formidable challenge. There are many variables to take into account, including duration of exposure, demography, geography, environment, and individual susceptibility. This is why the incidence of cancer attributable to exposure to toxic chemicals has been estimated to be between 1 and 19% (Anand et al., 2008; Colditz and Wei, 2012; Forouzanfar et al., 2015; IARC, 2018; Kessler, 2014; President's Cancer Panel, 2010).

General estimates from a recent WHO report (Prüss-Ustün et al., 2016) attribute approximately 20% of all cancers to environmental factors, with occupational exposure ranging between 2 and 8% and cancers due to chemicals in the environment around 1.5–2%.

Differences in estimates can be due to study context (target cancer or target pollutant) or to uncertainties in the collection of data or because of different approaches to data analysis. For example, the contribution of air pollutants to lung cancer has been attributed as follows: 17% to household air pollution, 14% to ambient air pollution, 7% to residential radon, 7% to occupational exposure, and 2% to second-hand tobacco smoke (Prüss-Ustün et al., 2016).

Moreover, as recently discussed at a Scientific Committee Seminar organized by the European Environmental Agency, there has been an overall underestimation of the potential risk of environmental chemicals, the majority of which are less studied. It has been reported in fact that there has been a historical bias toward deepening knowledge and providing more information on chemicals with known risk (EEA Chemicals for a sustainable future, 2018). The strongest evidence for cancer occurrence associated with chemical exposure is related to occupational settings. In the workplace, characterisation of the environment, exposure levels, exposure time-frame and the health status of workers can be tracked in a precise and accurate way. Acquisition of such data has led, for example, to the conclusion that lung cancer accounts for 54–75% of all occupational cancers (Cogliano et al., 2011; EU Commission Staff Working Document Impact Assessment, 2016).

### 3.1 Chemical carcinogens

Over the past 40 to 50 years, the International Agency for Research on Cancer (IARC) has classified over 1000 agents, with the majority being occupational chemicals and some complex mixtures. The evaluations have shown that 50% of them are truly, probably or possibly carcinogenic to humans, while the remaining 50% are not classifiable because of insufficient data (IARC, website). The chemical carcinogens identified by IARC largely overlap with those registered as carcinogens in the European Chemicals Agency (ECHA) inventory of harmonised classified substances (CLP Inventory, C&L), which represent about 3% (4604 out of 141,823, as on June 6, 2018) of the whole chemicals inventory (ECHA, website). This suggests that a significant number of chemicals are still in need of thorough review, implying a considerable demand for testing and evaluation of carcinogenic potential.

**Fig. 3:**
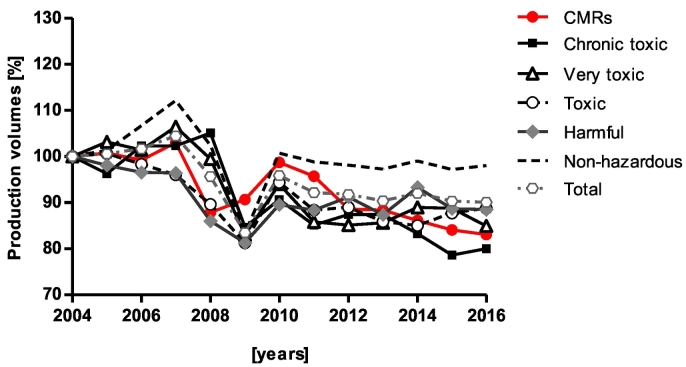
Chemical production volumes in the EU. Percent variation of aggregated production volumes, in million tons, of chemicals over years 2004–2016 in the EU. Chemicals were broken down into five toxicity classes and non-hazardous chemicals: carcinogenic, mutagenic and toxic to reproduction (CMR) chemicals; chronic toxic chemicals; very toxic chemicals; toxic chemicals; harmful chemicals and non-hazardous. These classes are derived from the risk phrases assigned to individual substances in Annex 6 of the Dangerous Substances Directive (amended in 2011), and adapted to the CLP most recent classification (EU Report, 2016). Data were elaborated from Eurostat (http://Ec.Europa.Eu/Eurostat/Web/Main/Home): as from 30/01/2018.

**Fig. 4:**
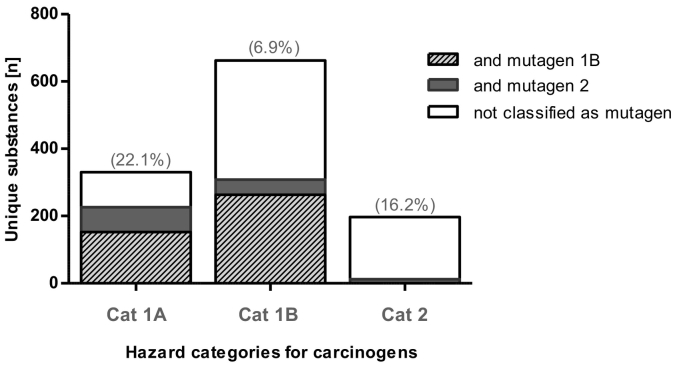
Substances classified as carcinogens. Data retrieved from the CLP inventory. Unique chemicals with harmonised classification for Carcinogenicity and Mutagenicity, based on CLP/GHS classification of hazard categories for carcinogens. The inventory contains classification and labelling information on notified and registered substances received from manufacturers and importers but it also includes the list of harmonised classifications. In parenthesis: percent (%) of chemicals with a classification for toxicity to reproduction within each carcinogen category. https://echa.europa.eu/information-on-chemicals/cl-inventory-database. Data were extracted on 03/08/2017.

The European Union deals on average with the production of >300 million tonnes of chemicals per year, of which 12–15% are classified as Carcinogenic, Mutagenic or toxic to Reproduction (CMRs, harmonised classification) (Eurostat; EU Report, 2016) (Fig. 3). These include more than a thousand chemicals known or presumed to be carcinogenic (Cat 1A and 1B) or suspected to be carcinogenic (Cat 2) and that can be predicted to induce mutagenic or toxic effects to reproduction (CLP Inventory, C&L) (Fig. 4). In this regard, the legislative system in place for registration and authorisation through REACH (EC Regulation 1907, 2006), and classification and labelling through the CLP Regulation (EC Regulation 1272, 2008), has contributed to the identification and management of the risks linked to the substances manufactured and marketed in the EU. In addition, several other pieces of EU legislation including those regulating biocides, pesticides, drinking water, and occupational safety and health (OSH) have all contributed to the stricter control of carcinogenic substances.

The implementation of the REACH and downstream sector-specific legislative measures (Supplementary Table 1) have led to a gradual decrease of production of highly toxic (chronic) and CMR chemicals (Fig. 3) (EC Communication, 2018; EU Report, 2016; EU Study, 2017). This reduction may be partly explained by the increase of chemicals identified as substances of very high concern (CMRs; Persistent, Bioaccumulative and Toxic substances - PBTs; very Persistent or very Bioaccumulative - vPvB) and classified as requiring either specific authorisation or restriction measures on marketing and use (Article 59(10)) (EC Regulation 1907, 2006). For example, the restriction of substances such as chromium (VI), dichloroethane, lead chromates, trichloroethylene and more recently decaBDE, PFOA, PFOA-related substances and PAHs has resulted in reduced risk for workers and consumers (EU Study, 2017).

A number of different health indicators are used to estimate the positive impact of chemical risk management such as reduced mortality, increased survival rates, reduction in direct and indirect medical costs and increases in worker productivity. These have shown that the strongest legislative impact has been primarily the reduction of occupational cancers, resulting from reduced exposure to occupational carcinogens by as much as 7% per year (EU Study, 2017). In relation to 13 well known carcinogens for example, it is estimated that over 1 million deaths from cancer have been avoided (EU Study, 2017). In general, therefore, one can assume that the risks of occupational exposure to carcinogens are well managed by all the legislative measures put in place over the last 20 years (Supplementary Table 1).

The assessment of the impact of EU chemicals legislation on environmental exposure of the general public has been limited by uncertainties related to data collection, health indicators and confounding factors. In addition, it is very difficult to describe the causal relationship between environmental chemicals and cancer disease (EU Report, 2016; EU Study, 2017). The assessments conducted also confirmed the need for more initiatives to generate information and facilitate understanding (e.g. via relevant indicators) concerning human exposure to environmental chemicals (e.g. from human biomonitoring programs), the carcinogenic properties of chemicals, and the contribution of chemical exposure to human disease.

Specific associations between chemical exposures and certain cancers have been recently reported. For example, breast cancer has been associated with the cumulative exposure to pesticides and other chemicals (Jeon et al., 2018; Rodgers et al., 2018). Several key studies have been reviewed, suggesting higher breast cancer risk for exposures during breast development to dichlorodiphenyltrichloroethane (DDT), dioxins, perfluorooctanesulfonamide (PFOSA) and air pollutants and, for occupational exposure, to solvents and other mammary carcinogens such as gasoline (Rodgers et al., 2018).

Approximately 5% of childhood cancers have also been estimated to result from environmental exposure to pollutants (Kessler, 2014; Prüss-Ustün et al., 2016; Roberts and Karr, 2012). To focus on one particular case in 2013, an eight-year-old Chinese girl was recognised as the youngest person to get lung cancer due to fine particulate matter via outdoor air pollution (Kessler, 2014), declared as carcinogenic to humans by IARC (IARC, 2016).

Concerns have also been reported concerning chronic, low-level exposure to chemical mixtures, which are considered to be poorly characterised and yet to be systematically addressed (Goodson et al., 2015). Notably, in recent years the European Commission, acknowledging that the assessment and management of mixtures is only partly covered by current legislation, has identified several gaps and areas for action. A number of initiatives both at research and policy level are on-going, which are expected to have a strong impact (EC Communication, 2012; Bopp et al., 2018a, Bopp et al., 2018b). Ongoing dialogue between the Commission services, European agencies and scientific experts is focused on reviewing the current state of knowledge and further elaborating and prioritising policy and research needs (Bopp et al., 2018a, Bopp et al., 2018b).

While some information on the effects of chemicals during critical windows of susceptibility (e.g. development, pregnancy or puberty) can be extrapolated from a number of available standard regulatory toxicity studies, no test method exists that involves exposure through the complete lifespan, from conception to old age, which also covers carcinogenicity assessment. Thus, effects undetected during those specific windows of exposure might still give concern for delayed effects leading to cancer later in life (such as for endocrine-disrupting chemicals or neuro- or immuno-developmental toxic chemicals) (Biro and Deardorff, 2013; Grandjean and Bellanger, 2017; Hughes and Waters, 2017; Osborne et al., 2015; Vandenberg et al., 2012). Moreover, individual substances can independently trigger several of the mechanisms of the carcinogenic process (EEA Chemicals for a sustainable future, 2018; Kienzler et al., 2016; Landrigan et al., 2017).

### 3.2 Changes in chemical exposure patterns

Recent discussions suggest that the REACH Legislation, while contributing to reducing the overall risks of chemicals in the environment, is protecting mainly against highly toxic chemicals in the workplace, and inadvertently encouraging the introduction of new substances with unknown properties (EEA Chemicals for a sustainable future, 2018; Scheringer, 2017).

Indeed, chemical exposure scenarios are changing quite rapidly both in terms of the amount and diversity of substances (Hendry et al., 2007) to which we are exposed. Changes in toxicological properties of chemicals and exposure patterns are predicted to adversely affect both human health and the environment (EEA, 2015). With regard to carcinogens, the proportion of non-genotoxic versus genotoxic carcinogens in the environment is expected to increase, since scientific knowledge on DNA reactivity allows industrial chemists to design compounds without overly reactive moieties. In addition, the manufacture and use of novel types of substances including nanomaterials, new generation pesticides and pharmaceuticals (e.g. biologicals, cell and gene therapies) are expected to increase. This raises new challenges for risk assessment and risk management. Testing procedures and regulatory information requirements will have to be revisited and gaps eventually filled.

### 3.3 Limitations of the current carcinogenicity assessment paradigm

Several groups have long questioned standard regulatory testing procedures which mainly rely on rodent assays (Table 1), pointing out a number of drawbacks related to their applicability to assess the potential of chemicals to cause cancer in humans (Heinonen et al., 2014; Knight et al., 2006; Paparella et al., 2017).

**Table 1:** Internationally agreed testing methods for carcinogenicity.

Test method	OECD test guideline	Species/number	Objective of the study	Duration of the study
Carcinogenicity studies	TG. 451	Rats and mice (50–65/sex/group) Non-rodents (mainly dog) (4–6/sex/group)	Observe test animals for a major portion of their life span for the development of neoplastic lesions during or after exposure to various doses of a test substance	Normally 24 months for rodents. For specific strains of mice, duration of 18 months may be more appropriate.
Combined chronic toxicity/ carcinogenicity studies	TG. 453	Rat (10/sex/group) chronic phase; and (50/sex/group) carcinogenicity phase	Identify carcinogenic and the majority of chronic effects and determine dose-response relationships following prolonged and repeated exposure.	Normally 12 months for the chronic phase, and 24 months for the carcinogenicity phase.
Chronic toxicity studies^a^	TG. 452	Rodents (20/sex/group) and non-rodents (4/sex/group)	Characterise the profile of a substance in a mammalian species following prolonged and repeated exposure.	Normally 12 months but, 6- or 9-month-studies are also performed.

Internationally agreed testing methods for carcinogenicity. Data were retrieved from the OECD website. The above methods are used by industry and governments for the regulatory safety testing of carcinogenic potential of chemicals.

a The chronic toxicity study is not aimed specifically at testing carcinogenicity, but it can be used for early detection of neoplastic lesions (Madia et al., 2016).

Scientific concerns include the overestimation of carcinogenic effects due to the typically high doses used in rodent studies and the uncertainties linked to extrapolation from rodent to humans due to species-specific biology and chemical mode of action (Cohen, 2017; Goodman, 2018; Madia et al., 2016). Importantly too, the 2-year rodent bioassay and chronic toxicity studies do not specifically address the four cancers of most concern described above since they were originally designed to cover a very wide range of possible health effects and cancer types. In addition, the 2-year rodent bioassay has proven inadequate to specifically predict hormonally induced reproductive tumours (Thayer and Foster, 2007). For some types of tumours such as prostate, no adequate animal model exists. In addition, for tumours in the ovary, the human tumours derive from different cellular origins than those induced by chemicals in rodents. In breast cancer, it has been reported that mammary gland premalignant lesions in mice do not parallel human pathological changes (Thayer and Foster, 2007).

Coupled with all these concerns over the scientific relevance of the animal tests for carcinogenicity, is the strong demand within the EU (EU Directive 63, 2010) to reduce the use of animals for scientific purposes and instead to use alternative (non-animal) approaches to fulfil regulatory testing requirements where possible.

Another important aspect of current practice is that carcinogenicity testing is rarely conducted under the REACH legislation unless triggered by specific alerts or exposure conditions (i.e. production volumes >1000 t/year (EC Regulation 1907, 2006); long-term exposure and widespread dispersive use; mutagens of category 3 or where there is evidence of hyperplasia or pre-neoplastic lesions from repeated-dose toxicity studies). This represents a potential protection gap, especially for non-genotoxic carcinogens that, when not classified for any other hazard property and not identified as such in (limited) repeated dose toxicity studies could go unidentified (EC Regulation 1907, 2006; ECHA R7a, 2017; Jacobs et al., 2016; Luijten et al., 2016; Madia et al., 2016).

In the case of cosmetic ingredients, for which in vivo testing is banned (EC Regulation 1223, 2009), the assessment of carcinogenicity relies on alternative testing approaches only. In the case of new ingredients that do not fall under other regulations, an in vitro genotoxicity test battery remains the main driver for carcinogenicity assessment. It is worth noting as well that for other sectors, such as pharmaceuticals, there is the proposal to waive the carcinogenicity rodent assay whenever sufficient supporting information is available (Braakhuis et al., 2018; Luijten et al., 2016; van der Laan et al., 2016).

## 4 Recommendations for adapting carcinogenicity assessment to meet future needs

The nature of cancer burden and the associated trends, together with changes in exposure patterns of chemicals in the environment need to be considered in anticipating how carcinogenicity assessment should evolve to offer adequate levels of protection to human health. The contribution of different risk factors, the prevalence of certain cancers over others, the evolution of the disease and the link to other morbidities, the exposure to chemicals in occupational or environmental settings, have all to be taken into account in devising cancer prevention strategies, which include a proper assessment of chemical carcinogenicity. Consequently, the approaches and test methods in use in regulatory toxicity testing need to be continuously adapted.

Initiatives are already underway to utilise new data- and knowledge-driven approaches in carcinogenicity assessment, which profit from the involvement and cooperation of both scientists and regulators from different product sectors. The integration of available information on relevant endpoints, including from epidemiology, traditional and alternative toxicology test systems, together with novel data streams, is undoubtedly considered a way forward to address in the short-term the limitations of the current carcinogenicity testing paradigm (Corvi et al., 2017).

Here, by broadening the context of regulatory toxicology to include more human specific cancer disease related-issues we emphasise a number of elements that we consider instrumental in providing some options to meet the future needs of carcinogenicity testing.

### 4.1 Addressing the four most prevalent cancers

One of the limitations of the current carcinogenicity testing paradigm, based on the 2-year rodent bioassay and chronic toxicity studies, is the difficulty to properly target the potential of a chemical to induce a specific type of cancer (Thayer and Foster, 2007). This is because the traditional animal studies were designed to identify any possible cancer. On the other hand, specific assessments on a routine basis for each cancer type are unlikely to be economically and practically feasible, given the huge number of chemicals in need of safety assessment.

As described above, recent cancer trends in mortality, incidence and prevalence (Bray et al., 2018; ECIS, 2018) reveal that cancers in breast, prostate, lungs and colon-rectum are the most prevalent. We therefore recommend prioritising the carcinogenicity assessment of chemicals for their potential to contribute specifically to the development of these four cancers. For this purpose, a number of options are available to address these specific cancers within toxicity testing strategies.

One option is to investigate the role of specific biomarkers that describe signalling pathways driving carcinogenesis in the tissues of interest for the four cancer types. The identification of signalling pathways that control cell progression, apoptosis or other common hallmarks of cancer are currently used to describe mechanisms and differences between individual tumours or tumour subtypes (e.g. oncogene and tumour suppressor proteins in breast cancer: Ras, c-Myc, p53; in colorectal cancer: Apc, Mlh1, Mlh2; or prostate cancer: Myc, Bcl-2, Hpn, PCA-3, P53 etc.). Such information is currently used in cancer research to identify causes of cancer and potential therapeutic targets (Sanchez-Vega et al., 2018) but could also be translated to toxicity studies.

A number of recently developed in vitro and in vivo cancer models, which provide detailed information on the mechanisms leading to the different cancers, can be used in regulatory toxicology. We recommend prioritising the development of models that detect specific traits of the most prevalent tumours (e.g., genetically engineered in vivo and advanced in vitro models). For example, cell culture methods for genetically predisposed breast or colon cancer are already being used in biomedical research (Janik et al., 2016; Telang and Katdare, 2007, Telang and Katdare, 2011). Also, 3D models (including organoids) are currently being investigated for their potential to accurately model physiology, shape and dynamics of colorectal and prostate cancers (Phillips, 2014; Vlachogiannis et al., 2018; Young and Reed, 2016).

Furthermore, biomarker gene signatures currently in use for early cancer diagnosis and clinical treatment decisions can be exploited to prioritise chemicals in need of thorough assessment for a specific cancer type. Grashow and co-workers (Grashow et al., 2018) have recently proposed an example of this type of approach. The authors have identified several occupational and environmental chemical classes that increase breast cancer risk. However, thousands of chemicals remain untested for their breast carcinogenic potential. Therefore, the authors have used biomarker gene kits from clinics to prioritise and curate a panel of genes which can serve as a biomarker of mammary toxicity and breast carcinogenesis (Grashow et al., 2018; Rodgers et al., 2018).

Similarly, gene signatures for sub-types of non-small cell lung cancer (NSCLC) (Shoshan-barmatz et al., 2017) have been recently described and might be used as biomarkers to screen for potential lung carcinogens. The advantage of using biomarker gene signatures lies in their applicability to different test systems: experimental in vivo or in vitro studies and in vitro High-Throughput Screening (HTS).

### 4.2 Better use of information on cancer aetiology and evolution in humans

A second option for adapting carcinogenicity assessment to evolving cancer scenarios is to make better use of knowledge of human physiology and pathophysiology deriving from research on human cancer biology, clinical studies and human biomonitoring. All these provide large amounts of relevant data that can inform toxicity studies.

Specific human relevant effects and events involved in the development of cancer disease in humans also represent important information. For example, immune effects, inflammatory events, epigenetic modifications, hormone alterations, including of the non-pituitary axis (e.g. Insulin Growth Factor 1, IGF-1) have been clearly identified as intermediate events in the carcinogenesis process (Jacobs et al., 2016; Martin, 2013; Villeneuve et al., 2018). New techniques and novel methods to accurately investigate such events are also becoming available and can be used to enhance standard toxicity studies (Smith et al., 2016; Guyton et al., 2018). This means opportunities to design fit-for-purpose studies based on more human-relevant data.

In this direction, although not overcoming all the uncertainties of standard in vivo studies, the recent update of standard 28-day and 90-day repeated dose toxicity studies (OECD TG 407, 2008; OECD TG 408, 2018) and the inclusion of endocrine disruptor assessment into legislation represent another step toward an improved assessment of carcinogenicity. These initiatives are expected to have a significant impact on the identification of endocrine disruptors and eventually those chemicals with the potential to induce hormone-related cancers (ECHA and EFSA Guidance Document, 2018). A significant impact is also expected from the on-going OECD project related to the development of an integrated approach to testing and assessment (IATA) for non-genotoxic carcinogens based on the inclusion of more human-related effects (Jacobs et al., 2016).

As mentioned above, increased cancer risk has been associated with several chronic disorders such as cardiovascular disease, diabetes, chronic kidney disease, and pulmonary disease (Tu et al., 2017). These include metabolic disorders that share similar signalling pathways and risk factors with breast, colon and prostate cancer (Persano et al., 2015; Vineis and Wild, 2014). Information on the links with those diseases should also be taken into account to better design testing strategies and be able to discriminate between chronic effects that may or may not lead to cancer.

Interactions between chemical exposure and ethnic/cultural background/diet/life style may also play a role in the manifestation of various cancer types. For example, in addition to environmental carcinogen exposures, a hormone-mediated difference in susceptibility to breast cancer has been observed among US women of different ethnic backgrounds, with the Afro-American ethnic group being more susceptible. A number of studies have also described interactions in the development of cancer in migrant populations. Asians or South Americans moving to North America or Europe and acquiring westernised life styles have shown increased cancer susceptibility compared to their populations of origin; in this context epigenetic modifications seem to play a key role (Martin, 2013). Additionally, endocrine disrupting chemicals have been reported to initiate or exacerbate obesity, a cancer-predisposing condition (Karoutsou and Polymeris, 2012).

Research and human epidemiology studies have identified critical windows of susceptibility that can increase cancer risk (Moirano et al., 2017; Prins et al., 2008; Rudel et al., 2011), as well as other diseases or long-term toxicity effects (e.g. neurotoxicity effects). In the case of breast cancer for example, gestation, early childhood, puberty and pregnancy may represent windows of susceptibility to environmental insults (Gopalakrishnan et al., 2017; Rodgers et al., 2018; Rudel et al., 2011). However, current animal-based toxicity test guidelines do not allow the study of carcinogenicity within specific windows of susceptibility. One adaptation in the conduct of in vivo studies is represented by the choice of the appropriate age of the experimental animal. However, the use of this option is limited by the lack of human relevance and other uncertainties (Thayer and Foster, 2007; Gopalakrishnan et al., 2017; Rodgers et al., 2018). The use of recently developed in vitro models of human cancer stem cells (CSCs, iPSCs) which play a key role in tumour formation (Persano et al., 2015), can overcome such limitations by providing a means of capturing mechanisms of early cancer development and progression (Franco et al., 2016; Persano et al., 2015).

Finally, we recommend that information on cancer aetiology and evolution in humans should be also interpreted in light of the impacts of policies currently in place. Such policies include those relating to health, nutrition, obesity, smoking or drinking behaviours, and results from recent EU initiatives, such as those tackling breast or colorectal cancer or the European Joint Action Innovative Partnership for Action Against Cancer (ECIS, 2018; IPAAC, 2018). This information is crucial to better understand the evolution of the disease in the exposed population, to interpret the links with other diseases or cancer-predisposing conditions and potential toxic effects, and to determine the relative contribution of different risk factors (Martin, 2013). It also helps to identify the most prevalent specific endpoints or mechanisms that should be included in the carcinogenicity assessment.

### 4.3 Use of biomarkers of exposure and human biomonitoring

To address the continuous change of chemicals present in the environment and evolving exposure scenarios, we recommend furthering the identification and use of biomarkers of exposure, effect and susceptibility. Biomarkers give evidence of association between exposure to specific chemicals and a carcinogenic effect and may provide information on mode of action. They provide a range of possible measurements from systemic exposure to resulting causal events in the process of carcinogenesis. The use of biomarkers has been recommended by the UK Committee on Carcinogenicity to establish recent exposures to actual or potential carcinogens not only in humans but also in experimental animals (Committee Carcinogenicity UK, 2013).

Biomarkers of exposure, referring to chemicals or metabolites measured in human biological media, provide a valuable means of tracking exposure levels in the general population and in subgroups with unusual exposures or vulnerabilities to certain diseases including cancer. Biomarkers of exposure can approximate internal dose and identify highly exposed groups (Rudel et al., 2014). Data from biomonitoring and biomarkers of exposure can be used as reference-values in risk assessment or guide the screening of potentially bio-accumulating chemicals (Neveu et al., 2017; Wild et al., 2013). Also, even if the exposure in occupational settings is typically higher than for consumers, it represents a relevant source of information on general chemical exposure effects and health impacts. In this context, the information gathered through the on-going European Initiative HBM4EU is expected to improve risk assessment by providing better evidence of the actual exposure of the population to chemicals, along with contextual information on possible health effects (HBM4EU, 2018). In fact, human biomonitoring in Europe has relied on well-established national programs in a number of EU countries (Ganzleben et al., 2017). Human biomonitoring data measurements are not currently required by the European Chemicals Legislation (EC Regulation 1907, 2006; ECHA R14, 2016; ECHA R15, 2016). However, such information is accepted and considered to give added value in the exposure assessment both for workers and consumers.

Biomarkers of exposure can also be used to monitor chemical combination (mixture) effects within changing chemical exposure scenarios. For example, a number of ongoing EU research projects are addressing research gaps in the area of mixture effects, including the development of joint epidemiological-toxicological approaches for mixture risk assessment and for prioritising mixtures of concern (Bopp et al., 2018a, Bopp et al., 2018b). Other projects are also expected to be valuable for carcinogenicity assessment. For example, the ChemDIS-Mixture tool can be used to identify potential effects of mixture interactions (Tung et al., 2018).

In addition, biomarkers of effect which can describe a key event implicated in a carcinogenic mode of action such as genotoxicity, changes in hormone levels, evidence of cell-specific toxicity (e.g. via altered proteins or altered gene expression) can be used to characterise the hazard and be considered as markers of adversity. For this purpose, the Adverse Outcome Pathway framework is an effective means to synthesise the relevant mechanistic knowledge in a form suitable for a regulatory context (Langley et al., 2015; Villeneuve et al., 2018).

Finally, furthering the investigation and use of biomarkers of susceptibility, acquired or inherited, which describe an individual's susceptibility to a specific cancer is important to better characterise the exposed population and eventually to identify individuals at high risk. For example, different genetic polymorphisms (e.g. overexpression of oncogenes or loss of function for tumour suppressor proteins) are used as biomarkers to identify individuals with a predisposition to develop breast cancer or colorectal cancer (Rothman, 2011). In addition, genetic susceptibility has been suggested to play a role in one's response to environmental chemical exposures. The investigation of gene-environment interaction can help to identify individuals at risk for specific chemical exposures. Preliminary evidence suggests for example that genetic variants in xenobiotic metabolism, DNA repair and immune response can modify the susceptibility to non-Hodgkin-lymphoma (NHL) following exposure to organochlorines, chlorinated solvents, chlordanes and benzene (Kelly and Vineis, 2014). This represents an important area of investigation with strong implications in terms of both chemical risk assessment and public health.

### 4.4 A practical approach toward modification of the current paradigm

The actual applicability of the options provided above is supported by a series of recent studies to evaluate chemicals for their potential to contribute to breast cancer risk (Grashow et al., 2018; Rodgers et al., 2018; Rudel et al., 2011; Schwarzman et al., 2015).

In the context of National Initiatives for New Chemicals Screening and Breast Cancer and Chemicals Policy Programs, a ‘disease endpoint’ approach is being developed. Starting from insights on the breast cancer aetiology and epidemiology evidence, a detailed analysis of biological effects of the disease and changes in biological pathways that serve as early indicators of toxicity has led to the development of tailored mechanistic tools able to identify specific breast cancer-inducing traits. Such new tools, such as the curated genomic biomarker panel for screening (Grashow et al., 2018) or the protocol for hazard identification (Schwarzman et al., 2015) can be fully integrated in the process of carcinogenicity assessment.

In this case, the public health concern of breast cancer incidence becomes the starting point to drive the risk assessment and to screen and prioritise chemicals specifically for that adverse effect. Hence, the knowledge of specific biological disease pathways drives the sorting of available and pertinent toxicity studies and where missing, the introduction of new data streams. This type of approach is suitable for screening and prioritising chemicals of concern in need of thorough assessment but is also applicable to different regulatory frameworks where for example, the hazardous properties of chemicals must be identified and reported on registration (e.g. REACH). Here, carcinogenicity information can be generated by the use of ad hoc studies, sorted on the basis of the hallmarks of cancer and description of the key characteristics of carcinogens and organized in the form of Integrated Approaches to Testing and Assessment (IATA) (Jacobs et al., 2016). We suggest therefore that such an approach represents an effective yet flexible means of carcinogenicity assessment that can accommodate different public health priorities and regulatory contexts.

## 5 Conclusions

The rising rates of cancer incidence and prevalence identified by the World Health Organization are of serious concern. The scientific advances of the past twenty years have helped to describe major properties of cancer disease, enabling therapies that are more sophisticated and effective. However, it has become clear that the management of relevant risk factors can also significantly reduce cancer occurrence worldwide. Public health policy actions cannot be decoupled from environmental policy actions, since exposure to chemicals through air, soil, water and food can contribute to cancer and other chronic diseases. Furthermore, due to the increasing global trend of chemical production including novel compounds, chemical exposure patterns are foreseen to change, posing increasing demands on chemical safety assessment, and creating potential protection gaps. The safety assessment of carcinogenicity needs to evolve to keep pace with changes in the chemical environment and cancer epidemiology. A number of tools are available or under development to more accurately assess the carcinogenicity endpoint. However, future strategies for assessing carcinogenicity should also take into account the prevalence of certain cancers, the contribution to the disease of different risk factors, the study of relationships between chemical exposure and risk factors, the disease aetiology and links with other disorders. In addition, changes in chemical exposure patterns and exposed populations are also critical considerations. A more holistic approach to carcinogenicity assessment would focus on the chemicals of highest concern, and use human-relevant testing methods to guide the most appropriate risk management measures.

The following is the supplementary data related to this article.

Supplementary Table 1 Main legislations and measures covering carcinogens in the EU. Supplementary Table 1

## Disclosure statement

Declarations of interest: none.
