# Supplementary material for: Carcinogenicity assessment: Addressing the challenges of cancer and chemicals in the environment
Source: Environ Int. 2019 Jul;128:417–29. doi: 10.1016/j.envint.2019.04.067 (PMC6520474; doi:10.1016/j.envint.2019.04.067)
Supplement: Supplementary Table 1 — Main legislations and measures covering carcinogens in the EU. [file mmc1.docx]

**Supplementary Material**

**Supplementary Table 1**

| **Supplementary Table 1.** **Main Legislations and measures covering carcinogens in the EU** | |
| --- | --- |
| **Legislations** | **Domain** |
| *Regulation EC 1907/2006 REACH* | Registration, Evaluation, Authorisation and Restriction of Chemicals |
| *Regulation EC 1272/2008 CLP* | CLP hazards of  substances and mixtures |
| *Directive 89/391 - OSH Framework Directive* | Safety and Health at Work |
| *Directive 2004/37/EC -(under revision)*  *COM(2018) 171 final* | Protection of workers from the risks related to exposure of carcinogens and mutagens at work Occupational exposure Levels (OELs) values |
| *Directive 98/24/EC (under revision)* | Chemical agents in the workplace |
| *Regulation EU 528/2012* | Biocides |
| *Regulation EU 283-284/2013* | Pesticides (PPPs) |
| *Regulation EC 1223/2009* | Cosmetic Ingredients |
| *EU Water Framework Directive 2000/60/EC* | Protection and enhancement of freshwater resources |
| *Drinking water Directive 98/83/EC (under revision)* | Limits for carcinogens in drinking water |
| *Directive2010/75/EU* | Solvents used in industrial environment |
| *Regulation EU 110/2011* | Materials in contact with food |
| *Seveso III Directive 2012/18/EU* | Industrial accidents |
